# The approximate functional equation of some Diophantine series

**DOI:** 10.1007/s00605-023-01859-6

**Published:** 2023-05-05

**Authors:** Fernando Chamizo, Bruno Martin

**Affiliations:** 1 Department of Mathematics, Universidad Autónoma de Madrid and ICMAT, Ciudad Universitaria de Cantoblanco, 28049 Madrid, Spain; 2 Laboratoire de Mathématiques Pures et Appliquées, CNRS, Université du Littoral Côte d’Opale, 50 rue F. Buisson, BP 599, 62228 Calais Cedex, France

**Keywords:** Diophantine series, Approximate functional equation, Convergence, Continuity, Primary 40A30, 11J70, Secondary 26A15

## Abstract

We prove that a family of Diophantine series satisfies an approximate functional equation. It generalizes a result by Rivoal and Roques and proves an extended version of a conjecture posed in their paper. We also characterize the convergence points.

## Introduction

Consider $g:\mathbb {R}\longrightarrow \mathbb {R}$ an odd 1-periodic $C^1$ function and $f:\mathbb {R}-\mathbb {Z}\longrightarrow \mathbb {R}$ a 1-periodic continuous function such that

1 $$\begin{aligned} L = \lim _{x\rightarrow 0}\Big (f(x)-\frac{\lambda }{x}\Big ) \end{aligned}$$

exists (and it is finite) for some $\lambda \ne 0$. This means that the only singularities of *f*, as a real function, are simple pole like singularities at the integers. For $(w,\alpha ) \in (\mathbb {R}^+)^2$, we introduce the Diophantine sum

2 $$\begin{aligned} \Phi _{{w}}(\alpha ) = \sum _{m=1}^{{\lfloor w \rfloor }} \frac{g(m^2\alpha )}{m^2} f(m \alpha ) \end{aligned}$$

where $\Phi _w(\alpha )$ is defined by continuity for $\alpha =p/q\in \mathbb {Q}$ with $q\le {w}$. Note that it makes sense because $g(x)\sim C(x-n)$ when $x\rightarrow n\in \mathbb {Z}$. Note also that with these assumptions, we have that $g(m^2\alpha )f(m \alpha )/m$ is uniformly bounded.

Motivated by a Diophantine approximation problem raised in [1], Rivoal and Roques proved in [2] that when $g(x)=\sin (2\pi x)$ and $f(x)=\cot (\pi x)$, $\Phi _{N}(\alpha )$ satisfies an approximate functional equation for $N\in \mathbb {Z}^+$. Namely, they show that

3 $$\begin{aligned} G_N(\alpha )= \Phi _N(\alpha ) -\alpha \Phi _{N\alpha }(1/\alpha ) - \frac{1}{\pi \alpha } \sum _{m=1}^N \frac{\sin (2\pi m^2\alpha )}{m^3} \end{aligned}$$

has a limit when *N* goes to infinity for each $\alpha \in (0,1]$, and that it is uniformly bounded in this interval. As a matter of fact, the last term could be replaced by $\log (1/\alpha )$. They conjecture that the limiting function is not only bounded but continuous. The existence of this limiting function contrasts with the fact, also proved for this choice of *g* and *f* in [2] (see also [1] for a weaker result), that $\lim _{N\rightarrow \infty }\Phi _N(\alpha )$ exists if and only if $\alpha$ is a Brjuno number, that is, an irrational number such that the convergents $p_n/q_n$ in its continued fraction satisfy $\sum q_n^{-1}\log q_{n+1}<\infty$.

As an aside, if we choose formally *g* as a constant, even replacing $n^2$ by $n^s$, approximate functional equations of similar kind (very explicit ones in some cases) can be established, although the convergence conditions are tighter (see [3–5]). These variants are related to some formulas of Ramanujan for $\zeta$ at odd values (see [6]) and to previous works of Lerch (see [7]). For other references and a historical account on approximate functional equations, see [8, §3].

The proof of the existence of the limiting function given in [2] uses heavily the additive properties of the sine function and the partial fraction expansion of the cotangent. We show here that this approximate functional equation also holds with the general definition of $\Phi _N$ as above and that it can be deduced from a mainly combinatorial argument not depending on the choice of *f* and *g*. We also prove the continuity of the limiting function for $\alpha >0$ and its continuous extension to $[0,\infty )$, settling in particular the conjecture posed in [2]. In the last section we apply a general result of [8] for certain approximate functional equations to characterize completely the convergence points of $\Phi _N$ when $N\rightarrow \infty$.

### Theorem 1

For $N\in \mathbb {Z}^+$ and $\alpha \in (0,1]$ consider

$$\begin{aligned} G_N(\alpha ) = \Phi _{N}(\alpha ) -\alpha \Phi _{N\alpha }(1/\alpha ) - T_N(\alpha ) \quad \text {with}\quad T_N(\alpha )= \frac{\lambda }{\alpha } \sum _{m=1}^N \frac{g(m^2\alpha )}{m^3} \end{aligned}$$

and $\Phi _N$ as in (2). Then the sequence of functions $\{G_N\}_{N= 1}^\infty$ is uniformly bounded on (0, 1] and it converges uniformly on each compact interval $I \subset (0,1]$ to a continuous function $G(\alpha )=O\big (\alpha \log (2\alpha ^{-1})\big )$.

### Remark

The limit of $T_N(\alpha )$ when $N \rightarrow \infty$ can be taken separately because the series converges but, as pointed out before, the separate existence of the limits of $\Phi _N(\alpha )$ and $\Phi _{N\alpha }(1/\alpha )$ is not assured in general (see Theorem 16). In [2], in the case $g(x)=\sin (2\pi x)$ and $f(x)=\cot (\pi x)$, the value $G(0)=0$ is implicitly assigned. We prefer here to let it undefined and to link it to the continuous extension through $G(0^+)=0$. Once we have stated the convergence in (0, 1] the extension to $\alpha > 0$ is rather easy.

### Corollary 2

If we let $\alpha \in \mathbb {R}^+$, $G(\alpha )=\lim G_N(\alpha )$ defines a continuous function on $\mathbb {R}^+$ satisfying $G(\alpha )=O\big (\alpha \log (\alpha ^{-1}+\alpha )\big )$. In particular $G(0^+)=0$ and *G* extends to a continuous function on $[0,\infty )$.

**Notation.** Given a real number *x*, $\Vert x\Vert$ stands for the distance from *x* to its nearest integer. Vinogradov’s notation $A\ll B$ is employed here with the same meaning as $A=O(B)$.

## Some reductions

We begin with some trivial remarks that eventually lead to some reductions in the proofs of Theorem 1 and Corollary 2.

### Lemma 3

If $\lambda$ is as in (1), the series

$$\begin{aligned} \sum _{m=1}^\infty \frac{g(m^2\alpha )}{m^2} \Big ( f(m \alpha ) - \lambda \pi \cot (\pi m\alpha ) \Big ) \end{aligned}$$

converges uniformly to a continuous bounded function on $\mathbb {R}$.

### Proof

We know that $f(x)-\lambda /x=O(1)$ and $\pi \cot (\pi x)-1/x=O(x)$ for $x\rightarrow 0$. Then $f(x)-\lambda \pi \cot (\pi x)$ is bounded in a neighborhood of 0 and, by the 1-periodicity, it is bounded on $\mathbb {R}$. $\square$

### Lemma 4

Assume that $\Omega (\alpha ) = \lim _{N\rightarrow \infty } \big ( \Phi _N(\alpha ) -\alpha \Phi _{N\alpha }(1/\alpha ) \big )$ is well defined for a given $\alpha >0$. Then $\Omega (1/\alpha )$ is also well defined and we have $\Omega (\alpha )+\alpha \Omega (1/\alpha )=0$.

### Proof

It follows easily from the definition of $\Omega (\alpha )$.

### Lemma 5

For $f(x)=\cot (\pi x)$ we have

$$\begin{aligned} \Phi _N(\alpha ) = \sum _{m=1}^N \frac{g(m\Vert m\alpha \Vert )}{m^2} f(\Vert m\alpha \Vert ). \end{aligned}$$

### Proof

If $\Vert m\alpha \Vert =\delta$, there exists $n\in \mathbb {Z}$ such that $m\alpha -n=\pm \delta$. Then $f(m\alpha )=f(\pm \delta )$ and $g(m^2\alpha )=g(mn\pm m\delta )={g}(\pm m\delta )$. When taking the product, the signs cancel because both functions *f* and *g* are odd. $\square$

The first two reductions and some simple manipulations with $T_N$ are enough to conclude Corollary 2. We abbreviate $\lim _{N\rightarrow \infty }T_N$ as *T*.

### Proof of Corollary 2

By Lemma 3, it is enough to consider the case $f(x)=\cot (\pi x)$. Theorem 1 assures the existence and the continuity of *G* in (0, 1] which are reflected into $[1,\infty )$ thanks to Lemma 4. The continuous extension to $[0,\infty )$ follows from the bound $G(\alpha )=O(\alpha \log (2\alpha )^{-1})$. The only missing point is the bound $G(\alpha )=O(\alpha \log \alpha )$ for large $\alpha$. By Lemma 4, for $\alpha >0$,

$$\begin{aligned} \alpha ^{-1}G(\alpha )= - G(\alpha ^{-1})-\alpha ^{-1}T(\alpha )- T(\alpha ^{-1}). \end{aligned}$$

By Theorem 1, $G(\alpha ^{-1})\rightarrow 0$ when $\alpha \rightarrow \infty$ and $\alpha ^{-1}T(\alpha )\rightarrow 0$ is obvious. Finally, since *g*(*x*) and *g*(*x*)/*x* are bounded, we have

$$\begin{aligned} \alpha \sum _{m=1}^\infty \frac{g(m^2/\alpha )}{m^3} \ll \alpha \sum _{m\ge \sqrt{\alpha }} \frac{1}{m^3} + \sum _{m<\sqrt{\alpha }} \frac{1}{m} \ll 1+ \log \alpha , \end{aligned}$$

showing that $T(\alpha ^{-1})=O(\log \alpha )$ when $\alpha \rightarrow \infty$. $\square$

## Proof of the main result

The key argument to prove Theorem 1 is that the terms in $\alpha \Phi _{N\alpha }(1/\alpha )$ are almost completely canceled by the terms in $\sum _{m=1}^{N} \frac{g(m\Vert m\alpha \Vert )}{m^2} f(\Vert m\alpha \Vert )$ such that $m\alpha$ is close to a positive integer. The following elementary result will be used to identify precisely these integers *m*. We remind the reader that if $y\in \mathbb {R}$ is not a half-integer, then $\lfloor y +1/2\rfloor$ gives the nearest integer to *y*. Note that for any $y\in \mathbb {R}$, $y = \lfloor y + 1/2 \rfloor \pm \Vert y\Vert$.

### Lemma 6

For $\alpha \in (0,1]$ fixed, the map $r_\alpha :\mathbb {Z}^+\longrightarrow \mathbb {Z}^+$ given by the formula $r_\alpha (n)=\lfloor n/\alpha +1/2\rfloor$ is injective and for $m\in \text { Im}\, r_\alpha$ it can be inverted with $r_\alpha ^{-1}(m) = \lfloor m\alpha +1/2\rfloor$.

### Proof

We can write $m = r_\alpha (n)$ as $m+\delta =n/\alpha +1/2$ for some $0\le \delta <1$. Eliminating *n* we have $n=m\alpha + (\delta -1/2)\alpha$ and so *n* is the nearest integer to $m\alpha$ because $\vert (\delta -1/2)\alpha \vert <1/2$ (the case $(\delta ,\alpha )=(0,1)$ cannot occur since it would imply the relation $m=n \pm 1/2$). Consequently, $n= \lfloor m\alpha +1/2\rfloor$. This proves that $r_\alpha$ is injective, hence the conclusion. $\square$

An important, and still elementary, remark is that although $r_\alpha$ may not map $[1,N\alpha ]\cap \mathbb {Z}$ onto $[1,N]\cap \text { Im}\, r_\alpha$, the surjectivity only may fail for one element: if $N=\lfloor n/\alpha +1/2 \rfloor$ with $n\in \mathbb {Z}^+$, *n* may not belong to $[1,N\alpha ]$.

### Lemma 7

For $N\in \mathbb {Z}_{>1}$ and $\alpha \in (0,1]$, let $N^*=N$ if $r_\alpha ^{-1}([1,N])\subset [1,N\alpha ]$ and $N^*=N-1$ otherwise. Then

$$\begin{aligned} r_\alpha :\, [1,N\alpha ]\cap \mathbb {Z}\longrightarrow [1,N^*]\cap \text { Im}\, r_\alpha \end{aligned}$$

is a bijection.

### Proof

Lemma 6 assures the injectivity. Clearly $r_\alpha ([1,N\alpha ]) \subset [1,N]\cap \mathbb {Z}$, so if $N^*=N$ the well-definition and the surjectivity are obvious. The case $N^*=N-1$ occurs if and only if $r_\alpha ^{-1}(N)>N\alpha$, equivalently, if and only if $N\alpha =\lfloor N\alpha \rfloor +\delta$ with $1/2\le \delta <1$. Hence $r_\alpha \big (\lfloor N\alpha \rfloor \big )=\lfloor N-\delta /\alpha +1/2\rfloor \le N^*$ and the map is well-defined. The surjectivity is obvious. $\square$

In the sequel, $\psi$ is a fixed 1-periodic continuous even function such that $0\le \psi \le 1$, $\psi (x)=1$ for $x\in [-1/4,1/4]$ and $\psi (x)=0$ for $x\in [3/8,5/8]$.

In the following result, we quantify the cancellation phenomenon described above between $\alpha \Phi _{N\alpha }(1/\alpha )$ and $\Phi _{N}(\alpha )$. It is the main responsible for the very existence of an approximate functional equation.

### Lemma 8

Given $n\in \mathbb {Z}^+$, consider $f_n:(0,1]\longrightarrow \mathbb {R}$ given by

$$\begin{aligned} f_n(\alpha ) = \frac{n^2}{m^2} g(m\Vert m\alpha \Vert )\cot (\pi \Vert m\alpha \Vert ) - \alpha g(n\Vert n/\alpha \Vert )\cot (\pi \Vert n/\alpha \Vert ) \end{aligned}$$

with $m = r_\alpha (n)$. Then $f_n(\alpha )=O(\alpha )$ with an *O*-constant only depending on *g*. Moreover, $\psi (n/\alpha )f_n(\alpha )$ is continuous and bounded on (0, 1].

### Proof

Let us abbreviate $\delta = \Vert m\alpha \Vert$. By Lemma 6, $n = \lfloor m\alpha + 1/2 \rfloor$, then we have $m-n/\alpha = \pm \delta /\alpha$ and, since $m= \lfloor n/\alpha +1/2 \rfloor$, we have $\Vert n/\alpha \Vert =\delta /\alpha$. It follows that $f_n(\alpha )=n^2\,g(m\delta )\cot (\pi \delta )/{m^2} - \alpha g(n\delta /\alpha )\cot (\pi \delta /\alpha )$. Using the estimate $\alpha \cot (\pi \delta /\alpha )- \alpha ^2\cot (\pi \delta )=O(\delta )$, we obtain

$$\begin{aligned} f_n(\alpha ) = \alpha ^2\cot (\pi \delta ) \Big ( \frac{\alpha ^{-2}n^2}{m^2} g(m\delta ) - g(n\delta /\alpha ) \Big ) +{O(\delta )}. \end{aligned}$$

The relation $m-n/\alpha = \pm \delta /\alpha$ implies $\alpha ^{-2}n^2/m^2=1+O\big (\alpha ^{-1}m^{-1}\delta \big )$. Since *g* is $C^1$ and periodic, we also have $g(m\delta )-g(n\delta /\alpha )\ll \vert m-n/\alpha \vert \delta \ll \delta ^2/\alpha$. These bounds give

$$\begin{aligned} f_n(\alpha )\ll \alpha ^2\delta ^{-1}\big (\alpha ^{-1}\delta ^{2}+\alpha ^{-1}m^{-1}\delta \big )+\delta \ll \alpha \end{aligned}$$

as expected.

For the continuity, note that $f_n$ is a composition of continuous functions except for the function $\alpha \mapsto r_\alpha (n)$ giving *m*, which is discontinuous when $n/\alpha$ is a half-integer. These discontinuities are eliminated by the factor $\psi (n/\alpha )$ because $\psi$ vanishes at the half-integers. $\square$

### Lemma 9

Given $m\in \mathbb {Z}^+$, the function

$$\begin{aligned} h_m(\alpha )= {\left\{ \begin{array}{ll} 1 &{}\text {if }m\not \in \text { Im}\, r_\alpha ,\\ 1-\psi \big (\alpha ^{-1}\Vert m\alpha \Vert \big ) &{}\text {if }m\in \text { Im}\, r_\alpha \end{array}\right. } \end{aligned}$$

is continuous on (0, 1].

### Proof

By definition, $m\in \text { Im}\, r_\alpha$ means that $n/\alpha +1/2=m+\delta$ for some $0\le \delta <1$ and some $n\in \mathbb {Z}^+$ and this is equivalent to

$$\begin{aligned} \alpha \in \bigcup _{n=1}^{\infty } \Big ( \frac{2n}{2m+1},\frac{2n}{2m-1} \Big ] \cap (0,1]. \end{aligned}$$

Note that $\alpha \mapsto \psi \big (\alpha ^{-1}\Vert m\alpha \Vert \big )$ is continuous on (0, 1] as a composition of continuous functions. Hence, if we check that both cases in the definition of $h_m$ coincide at the boundary points $\alpha =2n/(2m+1)$ and $\alpha =2n/(2\,m-1)$ for $1\le n<m$ then $h_m$ is continuous by the pasting lemma. This is the same as checking that $\psi \big (\alpha ^{-1}\Vert m\alpha \Vert \big )$ vanishes at $\alpha =2n/(2m\pm 1)$. Writing $m\alpha =n\mp n/(2m\pm 1)$, we have $\alpha ^{-1}\Vert m\alpha \Vert = \alpha ^{-1}n/(2m\pm 1)=1/2$ and $\psi (1/2)=0$. $\square$

The proof of Theorem 1 is based on a rearrangement of the terms in $\Phi _{N}(\alpha ) - \alpha \Phi _{N\alpha }(1/\alpha )$ involving the functions $f_n$ and $h_m$ introduced in the previous lemmas. In the rest of this section we assume $f(x)=\cot (\pi x)$ and consequently $\lambda =1/\pi$ in the definition of $T_N$. We can do it without loss of generality by Lemma 3.

For $w\in [0,\infty )$ and $\alpha \in (0,1]$, we set

$$\begin{aligned} S^{(1)}_{w}(\alpha )&= \sum _{n=1}^{\lfloor w\rfloor } \psi (n/\alpha ) \frac{f_n(\alpha )}{n^2},\\ S^{(2)}_{w}(\alpha )&= \alpha \sum _{n=1}^{\lfloor w \rfloor } \big (1-\psi (n/\alpha )\big ) \frac{g(n\Vert n/\alpha \Vert )}{n^2} \cot (\pi \Vert n/\alpha \Vert ), \\ S^{(3)}_w(\alpha )&= \sum _{m=1}^{\lfloor w \rfloor } h_m(\alpha )\frac{g(m\Vert m\alpha \Vert )}{m^2} \cot (\pi \Vert m\alpha \Vert ) \end{aligned}$$

and we denote by $S^{(j)}(\alpha )$ the corresponding infinite series.

### Lemma 10

For *N*, $N^*$ and $\alpha$ as in Lemma 7, we have the decomposition

4 $$\begin{aligned} \Phi _{N^*}(\alpha ) - \alpha \Phi _{N\alpha }(1/\alpha ) = S_{N\alpha }^{(1)}(\alpha )-S_{N\alpha }^{(2)}(\alpha )+S_{N^*}^{(3)}(\alpha ). \end{aligned}$$

### Proof

Let us set $a_m=g(m\Vert m\alpha \Vert )\cot (\pi \Vert m\alpha \Vert )/m^2$ and write $a_m=b_m+c_m$ with $b_m=a_m$ if $m\in \text { Im}\, r_\alpha$ and $b_m=0$ otherwise. With this notation, according to Lemma 5, we have $\Phi _{{N^*}}(\alpha )={\sum _{m=1}^{N^*}( b_m +c_m)}$. Lemmas 8 and 7 show that

$$\begin{aligned} \sum _{m=1}^{N^*} b_m-\alpha \Phi _{N\alpha }(1/\alpha )&= \sum _{n=1}^{\lfloor N\alpha \rfloor } \frac{f_n(\alpha )}{n^2} = S_{N\alpha }^{(1)}(\alpha ) + \sum _{n=1}^{\lfloor N\alpha \rfloor } \Big (1-\psi \big ( \frac{n}{\alpha }\big )\Big )\frac{f_n(\alpha )}{n^2}\\&= S_{N\alpha }^{(1)}(\alpha ) -S_{N\alpha }^{(2)}(\alpha ) + \sum _{m=1}^{N^*} \Big (1-\psi \big (\frac{r_\alpha ^{-1}(m)}{\alpha }\big )\Big )b_m \end{aligned}$$

where in the last sum, abusing of the notation, we assume that the undefined values of $r_\alpha ^{-1}(m)$ when $m\not \in \text { Im}\, r_\alpha$ are ignored because $b_m=0$. Using the identity

$$\begin{aligned} m\alpha = r_{\alpha }^{-1}(m) \pm \Vert m\alpha \Vert \end{aligned}$$

for $m\in \text { Im}\, r_\alpha$ and recalling that $\psi$ is even and 1-periodic, the last sum is $\sum _{m=1}^{N^*}h_m(\alpha )b_m= S_{N^*}^{(3)}(\alpha )-\sum _{m=1}^{N^*}c_m$. $\square$

### Lemma 11

For each $j\in \{1,2,3\}$, the series $S^{(j)}(\alpha )$ converges normally to a continuous function on each compact interval $I\subset (0,1]$.

### Proof

It is enough to check that the three functions

$$\begin{aligned} \psi (n/\alpha ) f_n(\alpha ), \, \big (1-\psi (n/\alpha )\big )\cot (\pi \Vert n/\alpha \Vert ) \text { and } h_m(\alpha )\cot (\pi \Vert m\alpha \Vert ) \end{aligned}$$

are continuous and bounded on each compact interval $I\subset (0,1]$. For the first function, this is the content of Lemma 8. For the second, it follows from the fact that $\psi (x)=1$ when $\Vert x\Vert \le 1/4$. For the third, first note that when $\Vert m\alpha \Vert <\alpha /4$, we have $m\in \text { Im}\, r_\alpha$, because in that case we have $m = \lfloor n/\alpha +1/2\rfloor$ with $n=\lfloor m\alpha +1/2\rfloor$ because $n=\alpha m+\varepsilon$ with $\vert \varepsilon \vert <1/4$. Also when $\Vert m\alpha \Vert <\alpha /4$, $\psi \big (\alpha ^{-1}\Vert m\alpha \Vert \big )=1$ and $h_m(\alpha )=0$ by definition of $\psi$. Consequently, the continuity and the compact boundedness of the third function follow from Lemma 9. $\square$

### Lemma 12

For $$N\ge 1$$ and $$\alpha \in (0,1]$$,

$$\begin{aligned} S_{N\alpha }^{(j)}(\alpha ) \ll \alpha \quad \text {for }j=1,2 \qquad \text {and}\qquad S_N^{(3)}(\alpha ) - T_N(\alpha )\ll \alpha \log (2 \alpha ^{-1}). \end{aligned}$$

### Proof

By Lemma 8, we have $S_{N\alpha }^{(1)}(\alpha )=O(\alpha )$ and the same bound applies to $S_{N\alpha }^{(2)}(\alpha )$ because $\big (1-\psi (n/\alpha )\big )\cot (\pi \Vert n/\alpha \Vert )$ is uniformly bounded. It remains to prove that

$$\begin{aligned} \sum _{m=1}^N \bigg ( h_m(\alpha ) \frac{g(m^2 \alpha )}{m^2} \cot (\pi m\alpha ) - \frac{g(m^2 \alpha )}{\pi \alpha m^3} \bigg ) = O\left( \alpha \log (2\alpha ^{-1})\right) . \end{aligned}$$

For $m\le \alpha ^{-1}/2$, clearly $m\not \in \text { Im}\, r_\alpha$ and hence $h_m(\alpha )=1$. Using the estimate $\cot x-1/x=O(x)$, we obtain that the part of the sum corresponding to this range is $O(\alpha \log (2\alpha ^{-1}))$. On the other hand, in the complementary range $m> \alpha ^{-1}/2$, we have $\sum g(m^2\alpha )/m^3\ll \alpha ^2$ and it only remains to show that

5 $$\begin{aligned} \sum _{m> \frac{1}{2}\alpha ^{-1}} h_m(\alpha )\frac{\vert \cot (\pi m\alpha )\vert }{m^2} \ll \alpha \log \left( 2\alpha ^{-1}\right) . \end{aligned}$$

First note that if $\Vert m\alpha \Vert \le \alpha /4$, then $h_m(\alpha )=0$ (see the proof of Lemma 11). Now we have

$$\begin{aligned} \sum _{\begin{array}{c} m> \frac{1}{2}\alpha ^{-1}\\ \Vert m\alpha \Vert >\alpha /4 \end{array}} \frac{1}{m^2\Vert m\alpha \Vert } \ll \sum _{q=1}^\infty \frac{1}{(q\alpha ^{-1})^2} \sum _{k< \frac{1}{2}\alpha ^{-1}} \frac{1}{\alpha k}. \end{aligned}$$

This inequality follows by writing $m=q\lfloor \alpha ^{-1}/2\rfloor +r$ with $0\le r<\lfloor \alpha ^{-1}/2\rfloor$ and by noting that $\Vert m\alpha \Vert$ presents gaps of size $\alpha$ when *r* varies. The last double sum amounts $O(\alpha \log (2\alpha ^{-1}))$ which proves (5). $\square$

### Proof of Theorem 1

Recall that we have defined $T(\alpha )=\lim T_N(\alpha )$. The convergence is uniform, hence *T* is continuous on (0, 1]. On the other hand, the trivial bound gives $\Phi _N(\alpha )- \Phi _{N^*}(\alpha )\ll N^{-1}$. Hence, considering the decomposition (4), the uniform convergence of $G_N(\alpha )$ on every interval $[a,b]\subset (0, 1]$ to

6 $$\begin{aligned} G(\alpha )= S^{(1)}(\alpha ) - S^{(2)}(\alpha ) + S^{(3)}(\alpha )-T(\alpha ) \end{aligned}$$

follows from Lemma 11. The uniform boundedness of $G_N(\alpha )$ on (0, 1] and the bound $G(\alpha ) =O(\alpha \log (2 \alpha ^{-1}))$ are consequences of Lemma 12.

We can also state a result à la Hardy-Littlewood (cf. [9, Th. 2.128]). $\square$

### Proposition 13

For $N\ge 1$ and $\alpha \in (0,1]$, we have

$$\begin{aligned} \Phi _N(\alpha ) -\alpha \Phi _{N\alpha }(1/\alpha ) =T_N(\alpha ) +G(\alpha ) +O\big ( \min \big ( ( N\alpha )^{-1}, \alpha \log ( 2\alpha ^{-1})\big ) + N^{-1} \big ). \end{aligned}$$

### Proof

The trivial bound proves that $\Phi _{N}(\alpha )-T_{N}(\alpha )$ and $\Phi _{N^*}(\alpha )-T_{N^*}(\alpha )$ differ in $O(N^{-1})$ which is absorbed by the error term, then it is enough to prove that $\Phi _{N^*}(\alpha ) -\alpha \Phi _{N\alpha }(1/\alpha ) - T_{N^*}(\alpha ) -G(\alpha )$ is $\ll \alpha \log ( 2\alpha ^{-1})$ and $\ll (N\alpha )^{-1}$. Thanks to (4) and (6), it equals

$$\begin{aligned} \big (S^{(1)}_{N\alpha }(\alpha ) - S^{(1)}(\alpha )\big ) - \big (S^{(2)}_{N\alpha }(\alpha ) - S^{(2)}(\alpha )\big ) + \big (S^{(3)}_{N^*}(\alpha ) - T_{N^*}(\alpha )\big ) - \big (S^{(3)}(\alpha ) - T(\alpha )\big ) \end{aligned}$$

which is $\ll \alpha \log (2\alpha ^{-1})$ according to Lemma 12. On the other hand, arranging the last two terms as $\big (S^{(3)}_{N^*}(\alpha )-S^{(3)}(\alpha )\big ) +\big (T(\alpha )-T_{N^*}(\alpha )\big )$ and following the proof of Lemma 11, we have $S^{(j)}_{N\alpha }(\alpha )-S^{(j)}(\alpha ) \ll ( N\alpha )^{-1}$ for $j=1,2$, $S^{(3)}_{N^*}(\alpha )-S^{(3)}(\alpha ) \ll N^{-1}$ and trivially $T(\alpha )-T_{N^*}(\alpha )\ll N^{-2} \alpha ^{-1}$. $\square$

## Convergence of $\Phi _N$

If we slightly strengthen the regularity requirements for *g*, it is possible to characterize the convergence points of $\Phi _N$ when $N\rightarrow \infty$, extending [2, Th. 2]. Namely, we impose in this section $g\in C^{1,\gamma }$ for some $0<\gamma <1$. This means that $g'$ satisfies locally the Hölder condition $g'(y)-g'(x)=O\big (\vert y-x \vert ^\gamma \big )$. In fact it is enough to require it for $x=0$.

The characterization of the convergence points is a quite direct application of a general result in [8] on approximate functional equations (see [8, Prop. 2] and [8, §6.2]). The point to be checked is a simple analytic fact.

### Lemma 14

Let *g* be a bounded function in $C^{1,\gamma }$ for some $0<\gamma <1$ with $g(0)=0$. Then

$$\begin{aligned} \frac{1}{x} \sum _{m=1}^\infty \frac{g(m^2 x)}{m^3} - \frac{1}{2} g'(0)\log \frac{1}{x} \end{aligned}$$

is a bounded function on (0, 1].

### Proof

We can restrict the sum to $m<x^{-1/2}$ because the rest of the terms give a bounded contribution to the function by the trivial estimate. On the other hand, we know that $g'(\xi )-g'(0)=O(\vert \xi \vert ^\gamma )$ and the mean value theorem shows that $g(t)=g(t)-g(0)=g'(0) t +O\big (t^{\gamma +1}\big )$ for $0\le t<1$. Then

$$\begin{aligned} \sum _{m<x^{-1/2}} \frac{g(m^2 x)/(m^2 x)}{m} = \sum _{m<x^{-1/2}} \frac{g'(0)}{m} + O\Big ( \sum _{m<x^{-1/2}} x^{\gamma }m^{2\gamma -1} \Big ). \end{aligned}$$

The first sum is $g'(0)\log x^{-1/2}+O(1)$ and the second is *O*(1). $\square$

The previous result shows that the case $g'(0)=0$ is special and that in fact, the sum in Theorem 1 could be omitted. The series appearing in the next result is a model of what happens in this case under $g\in C^{1,\gamma }$. The purpose of introducing it here is to give a common treatment to $g'(0)\ne 0$ and $g'(0)= 0$ in the proof of Theorem 16 (in fact, the absolute convergence in the latter case can be obtained as a quite direct consequence of it). A secondary reason is to show that an extra factor $\Vert n^2x\Vert ^{\gamma }$ forces the convergence everywhere of the series in [1, §4] (recall that $\big \Vert n\Vert nx\Vert \big \Vert =\Vert n^2x\Vert$).

### Lemma 15

Let $\gamma >0$. The series

$$\begin{aligned} \sum _{n=1}^\infty \frac{\big \Vert n\Vert nx\Vert \big \Vert ^{\gamma +1}}{n^2\Vert nx\Vert } \end{aligned}$$

converges for every $x\in \mathbb {R}$ and determines a bounded function, where the terms with $\Vert nx\Vert =0$ are defined (by continuity) as 0.

### Proof

Of course, $\Vert nx\Vert =0$ only occurs if $x= a/q\in \mathbb {Q}$ with $q\mid n$. On the other hand, $\sum _{n=1}^{q-1} \Vert na/q\Vert ^{-1}\ll q\log q$ if $q\not \mid n$. Then subdividing the range of *n* into blocks of length *q*, the series for $x=a/q$ is bounded by $\sum _{k=1}^\infty (qk)^{-2}q\log q$, which converges and is uniformly bounded.

For $x\not \in \mathbb {Q}$, let *S* be the part of the series corresponding to $q\le n<Q$ with *p*/*q* and *P*/*Q* consecutive convergents in the continued fraction of *x*. It is enough to prove $S=O(q^{-\sigma })$ for some $\sigma >0$ because the denominators of the convergents grow exponentially [10, Th.12]. Replacing $\big \Vert n\Vert nx\Vert \big \Vert$ by 1, the terms in *S* with $q\not \mid n$ contribute $O\big (q^{-1}\log (2q)\big )$ as shown in the proof of [4, Prop.3.1] (where they are labeled $S_2$ and $S_3$) taking $k=1$, $s=2$. If $q\mid n$, [4, Lem. 2.3] proves that $n\Vert nx\Vert \le 1$ and $n\Vert nx\Vert \ge 1$ imply, respectively, $n\ll \sqrt{qQ}$ and $n\gg \sqrt{qQ}$ because $qQ\Vert nx\Vert \asymp n$. Summing up,

$$\begin{aligned} S \ll \sum _{\begin{array}{c} q\le n\ll \sqrt{qQ}\\ q\mid n \end{array}} \frac{(n^2/qQ)^{1+\gamma }}{n^2(n/qQ)} + \sum _{\begin{array}{c} \sqrt{qQ}\ll n<Q\\ q\mid n \end{array}} \frac{1}{n^2(n/qQ)} +\frac{\log (2q)}{q}. \end{aligned}$$

Writing $n=kq$, we obtain that each sum is $O(q^{-1})$. $\square$

Now, we are ready to decide about the convergence of $\Phi _N$ when $N\rightarrow \infty$. To keep the coherence with the previous parts, we state the result for positive numbers, but the periodicity allows to extend it to $\mathbb {R}$.

### Theorem 16

Let *f* and *g* as in the introduction but imposing also $g\in C^{1,\gamma }$ for some $0<\gamma <1$. For $x\in \mathbb {R}^+$ and $g'(0)\ne 0$, the series

$$\begin{aligned} \sum _{m=1}^{\infty } \frac{g(m^2x)}{m^2} f(m x) \end{aligned}$$

converges if and only if *x* is a Brjuno number. On the other hand, it converges for every $x\in \mathbb {R}^+$ if $g'(0)=0$.

### Proof

By the 1-periodicity of the series, we can restrict ourselves to $x\in (0,1]$. And we can also suppose that $f(x) = \cot (\pi x)$ (see Lemma 3). In this interval, the result for *x* irrational is an application of Proposition 2 in [8], which adresses approximated functional equations involving the Gauss map (denoted by $\alpha$ in [8])

$$\begin{aligned} T(x)= \frac{1}{x} \bmod 1. \end{aligned}$$

We denote by $T^k$ the iterates of *T* for $k\ge 1$. Now set

$$\begin{aligned} \mathcal {S}(x) = \sum _{j\ge 0} x T(x) \ldots T^{k-1}(x) (H+G)(T^k(x)), \end{aligned}$$

where *H*(*x*) is the function in Lemma 14 multiplied by $\lambda$. This infinite series corresponds to $\mathcal {S}_{G+H}(x)$ in [8] (see [8, (16)]). Note that $\mathcal {S}(x)$ converges for every irrational *x* because $G+H$ is bounded and $x T(x) \ldots T^{k-1}(x) \le F_{k+2}$ where $F_k$ stands for the *k*-th Fibonacci number (see [11, (11) and (19)]). Also we have $\mathcal {S}(x) -x \mathcal {S}(1/x)=(G+H)(x)$. We apply Proposition 2 in [8] with (we rename *g* there as $g^*$ to avoid the clash of notation) $a=\theta =s=1$, $f(x,v)=\Phi _v(x)-\mathcal {S}(x)$, $g^*(x)=\frac{1}{2} \lambda g'(0)\log (1/x)$. It states that if

$$\begin{aligned} \varepsilon (x,v)=f(x,v)- xf(1/x,xv)-g^*(x) \end{aligned}$$

Satisfies the four conditions

(i) for all $$x \in \, ]0,1[\,$$, $$\varepsilon (x,v) \rightarrow 0 \quad (v \rightarrow \infty )$$;

(ii) $$\;\varepsilon (x,v) \ll 1 \quad (0<x<1, \, x^a v \ge 1)$$;

( iii) $$f(x, 1) \ll 1 \quad (0<x<1)$$;

(iv) $$f(x, v) \ll 1 +\vert g^*(x)\vert \quad (0<x<1, \, x^av< 1)$$;

then for all irrational $x\in ]0,1[$, $\lim _{v \rightarrow +\infty } f(x,v)$ exists if and only if

7 $$\begin{aligned} \sum _{j\ge 0} x T(x) \ldots T^{j-1}(x) \log (T^j(x)) < \infty . \end{aligned}$$

Since (7) characterizes Brjuno numbers (see for instance [11, proposition 1]), the conclusion for irrational *x* will follow. Checking (i), (ii), (iii) and (iv) is very similar to the case $g(x)=\sin (2\pi x)$ and $f(x)=\cot (\pi x)$ studied in [8, §6.2]^1^. Set

$$\begin{aligned} \eta (x,v)= x\Phi _{x v }(1/x)- x\Phi _{x\lfloor v\rfloor }(1/x). \end{aligned}$$

and extend the definition of $G_N$ by $G_v = G_{\lfloor v\rfloor }$ for $v\ge 1$. We have

$$\begin{aligned} \varepsilon (x,v) = G_v(x)-G(x)-\frac{\lambda }{x} \sum _{m>v} \frac{g(m^2x)}{m^3}-\eta (x,v), \end{aligned}$$

and since $$\eta (x,v) \ll x\sum _{x\lfloor v\rfloor < n\le x v} n^{-1}\ll v^{-1},$$

$$\begin{aligned} \varepsilon (x,y) = G_v(x)-G(x)+O(x^{-1}v^{-2}+v^{-1}). \end{aligned}$$

Conditions (i) and (ii) follow from Theorem 1. The condition (iii) is obvious in our case. The condition (iv) requires $\Phi _v(x)\ll \log (v+2)$ if $g'(0)\ne 0$ and $\Phi _v(x)\ll 1$ if $g'(0)= 0$. The first case follows $g(m^2x)f(mx)\ll g(m\Vert m x\Vert )/\Vert mx\Vert \ll m$ and the second case, using $g(t)=O\big (\vert t \vert ^{\gamma +1}\big )$, follows from $g(m^2x)f(mx)\ll \big \Vert m\Vert m x\Vert \big \Vert ^{\gamma +1}/\Vert mx\Vert$ and Lemma 15.

It remains to consider $x=a/q\in \mathbb {Q}^+$. Lemma 15 covers the case $g'(0)=0$ as before. If $g'(0)\ne 0$ it is easy to check that it does not converge. Note that $g(m^2x)f(mx)$ tends to $\lambda g'(0)m$ when $q\mid m$ and remains bounded otherwise giving a divergence as in the harmonic series (see [2, p.98]). $\square$

## Data Availability

Data sharing not applicable to this article as no datasets were generated or analysed during the current study.

## Footnotes

1 Note however an imprecision in [8, §6.2]: the equation (43) which comes from [2] holds *a priori * only for integer values of *v*.
